# Insights into Pharmacists’ Participation in Professional Certification Programs in Saudi Arabia

**DOI:** 10.3390/healthcare12191943

**Published:** 2024-09-28

**Authors:** Ammar Y. Alhuzli, Mohammed B. Alzahrani, Ahmed J. Althobaiti, Abdullah S. Alshammari, Adnan Alharbi, Mahmoud Elrggal, Nasser M. Alorfi, Foud O. Bahamdain, Walaa Alnemari, Mohammed Alrashed, Abdulmalik S. Alotaibi, Mohammed A. Alnuhait

**Affiliations:** 1 Pharmaceutical Practices Department, College of Pharmacy, Umm Al-Qura University, Makkah 24381, Saudi Arabia; s441005579@st.uqu.edu.sa (A.Y.A.); s441008482@st.uqu.edu.sa (M.B.A.); s441004324@st.uqu.edu.sa (A.J.A.); asshammari@uqu.edu.sa (A.S.A.); assharbi@uqu.edu.sa (A.A.); assalotaibi@uqu.edu.sa (A.S.A.); 2 Pharmacology and Toxicology Department, Faculty of Medicine, Al Qunfudah, Umm Al-Qura University, Makkah 21961, Saudi Arabia; merggal@uqu.edu.sa; 3 Pharmacology and Toxicology Department, College of Pharmacy, Umm Al-Qura University, Makkah 24381, Saudi Arabia; nmorfi@uqu.edu.sa; 4 Pharmaceutical Care Services, King Abdullah Medical City, Makkah 21955, Saudi Arabia; bahamdain.f@kamc.med.sa; 5 Department of Community Medicine, Faculty of Medicine, Umm Al-Qura University, Makkah 24382, Saudi Arabia; wdnemari@uqu.edu.sa; 6 Department of Pharmacy Practice, College of Pharmacy, King Saud bin Abdulaziz University for Health Sciences, Riyadh 11841, Saudi Arabia; alrashidm@ksau-hs.edu.sa; 7 King Abdullah International Medical Research Center, Riyadh 11481, Saudi Arabia; 8 Department of Pharmaceutical Care Services, King Abdulaziz Medical City, Riyadh 14611, Saudi Arabia

**Keywords:** pharmacist, certification, Saudi Arabia, professional development, programs

## Abstract

Introduction: Professional certification programs play a crucial role in helping pharmacists develop specialized skills and establish their expertise in the field. This study explores how pharmacists in Saudi Arabia engage with these programs by examining their levels of awareness, attitudes, and participation. It also identifies the key barriers and motivators that influence their decision to pursue certification. Methods: An online survey was conducted among pharmacists in Saudi Arabia to assess participants’ knowledge of certification programs, their perceived benefits, the actual participation rates, and their preferences regarding certification areas and assessment methods. Results: Out of 394 participating pharmacists, around 47% had never participated in a professional certification program, and about 27% of our sample were unaware of such programs in Saudi Arabia. Financial constraints and a lack of awareness were noted as the primary barriers to participation. Almost 55% showed an interest in Clinical Pharmacy Specialties certification, while 54% were inclined toward Pharmacy Management and Leadership certifications. A preference for practical assessments in certification programs was suggested by 55% of participants. Conclusions: This study highlights a significant need for increased awareness and accessibility to professional certification programs among pharmacists in Saudi Arabia. Addressing participation barriers is vital to foster professional development and meet the healthcare sector’s evolving demands in Saudi Arabia.

## 1. Introduction

Professional certification represents formal recognition confirming that an individual meets set standards in the knowledge, training, and skills necessary for a specific medical or professional practice. These certifications are issued by governing bodies like professional societies, universities, or private agencies or organizations, often comprised of experienced practitioners who are typically certified themselves. These organizations operate independently and are responsible for all critical decisions regarding certification processes [1]. Over recent decades, there has been a significant rise in credentialism across various sectors, particularly in healthcare, where a high percentage of workers hold these credentials. Historically, credentialing was predominantly associated with degree-holding professions such as nursing, medicine, and pharmacy. Professional organizations have actively developed credentials to boost their members’ knowledge, skills, and wages, especially targeting workers with standardized skill sets. Some certifications are valid for a limited time and need periodic renewal, while others are valid indefinitely, provided that ongoing educational requirements are met [2]. For example, the Board of Pharmacy Specialties (BPS) offers certifications to pharmacists both in the U.S. and internationally in various areas. These specialties include ambulatory care, cardiology, sterile compounding, critical care, geriatrics, infectious diseases, nuclear pharmacy, nutrition support, oncology, pediatrics, pharmacotherapy, psychiatric pharmacy, and solid organ transplantation [3]. While academic institutions are developing specialized degrees in pharmacy programs, including degrees such as Bachelor of Pharmacy, Master of Pharmacy, and Doctor of Pharmacy, along with various postgraduate options, this expansion alone may not be sufficient. In addition to international certifications, the Saudi Society of Clinical Pharmacy (SSCP) provides local professional certifications for Saudi pharmacists, such as the Medication Therapy Management (MTM) professional certificate and the Sterile Product Preparation Professional Certification, which both support the advancement of pharmacy practice in Saudi Arabia [4]. It is important to differentiate between the Pharm-D and B.Pharm programs. The Pharm-D is typically a 6-year program, designed to be more clinically oriented with a focus on direct patient care, while the B.Pharm is generally a 4- or 5-year program that emphasizes pharmaceutical sciences and theoretical knowledge [5]. The evolving landscape of the pharmaceutical sector increasingly emphasizes professional certifications, reflecting a shift toward more practical, patient-focused training. This trend underscores the necessity for pharmacists to continuously enhance their skills and knowledge through structured certification programs, ensuring their alignment with the dynamic requirements of modern healthcare [5,6]. It is also important to consider that Saudi Arabia’s workforce is diverse, with pharmacists of various nationalities, particularly from developing countries, contributing significantly to the healthcare system [7]. Understanding their perspectives on professional certification is crucial, as their willingness or interest in such certifications may vary depending on their background and experience. Employers play a pivotal role in encouraging or discouraging certification uptake, depending on whether they provide financial assistance, time off, or recognition for certified pharmacists. Pharmacy’s role has expanded beyond traditional dispensing, placing pharmacists at the forefront of patient-centered care and public health services. To deliver this effectively, they must continuously enhance their skills and knowledge. Continuing education and certification are vital for this development [6,8]. In our study, “professional certification” is defined as the formal accreditation that pharmacists earn after completing advanced training or passing specific exams in a specialized area of their field. This encompasses various types of credentials, such as board certifications or specialized certificate programs, which go beyond standard licensing. These certifications are aimed at improving professional competency, advancing career prospects, and enhancing the quality of care provided by pharmacists in their respective areas of practice [9,10]. Pharmacists in Saudi Arabia are required to participate in continuing education programs, as mandated by the Saudi Commission for Health Specialties (SCFHS), to maintain their licensure and stay updated on advancements in pharmacy practice [11]. This study aims to address a significant gap in the current literature by thoroughly analyzing the awareness, attitudes, and participation of pharmacists in professional certification programs in Saudi Arabia. This study’s objective is to evaluate pharmacists’ understanding and attitudes toward certification, examining their participation levels and the key factors motivating them to pursue these credentials.

## 2. Methods

### 2.1. Study Design

This study utilized a cross-sectional survey to investigate pharmacists’ awareness, attitudes, and participation in professional certification programs in Saudi Arabia. The survey examined pharmacists’ knowledge of available programs, evaluated their attitudes and perceived benefits or barriers, and measured their participation rates. The survey was conducted electronically over a one-month period in November 2023 and was distributed via email lists from licensing authorities, as well as through social media platforms such as LinkedIn, Twitter, and Facebook. It targeted pharmacists working in various healthcare settings across the country. The inclusion criteria required participants to be licensed pharmacists currently practicing in Saudi Arabia. We hypothesized that there would be limited interest and willingness among pharmacists in the pharmaceutical sector to participate in professional certification programs. Additionally, we anticipated that knowledge gaps and a lack of institutional support may hinder the uptake of these certifications. We estimated that awareness of professional certification programs among our target population would be around 50%. To calculate the appropriate sample size, we assumed a precision level of 5%, a 95% confidence interval, and a pharmacist population of approximately 30,840, based on data from the Ministry of Health and the Saudi Commission for Health Specialties [7]. This calculation yielded a sample size of 380 participants. Convenience sampling was utilized for participant recruitment.

### 2.2. Questionnaires and Data Collection

The cross-sectional survey was designed to explore the awareness, attitudes, and engagement of pharmacists in Saudi Arabia in professional certification programs. The survey instrument was carefully developed and validated through an extensive review of the relevant literature, alongside consultation with a panel of five senior pharmacy experts, each with over 10 years of experience in clinical practice, pharmacy education, and certification, to ensure the accuracy and relevance of the questions. We sought input from a panel of experts in pharmacy education and professional development to ensure the questions were relevant, clear, and comprehensive. The survey consisted of 25 questions, organized into sections that each addressed a specific aspect of the study objectives. These sections included demographic information, awareness of certification programs, motivations for participation, perceived benefits, barriers to certification, and willingness to engage in such programs. To ensure that the survey was effective, we conducted a pilot test with 10 pharmacists from various practice settings. These pharmacists completed the survey and provided valuable feedback regarding the clarity of the questions, the relevance of the content to their professional experiences, and the overall user-friendliness of the survey. This process enabled us to assess both face validity, ensuring that the survey appeared to measure what it was intended to measure, and content validity, confirming that it comprehensively covered the key aspects of professional certification. Their insights were instrumental in refining the survey, making it clearer and more accessible to a broader group of pharmacists.

Participation in this study was restricted to licensed pharmacists actively practicing in Saudi Arabia. Data collection occurred over a four-week period, during which responses were continuously monitored to ensure that the sample remained representative. Afterward, the data were carefully cleaned and coded for analysis.

### 2.3. Ethical Considerations

This study was conducted in accordance with ethical guidelines, and approval was obtained from the Umm Al-Qura University Ethics Committee (Approval Number: HAPO-02-K-012-2023-10-60923). All participants provided informed consent before taking part in the survey, and their responses were anonymized to protect their privacy.

### 2.4. Statistical Analysis

The data were analyzed using Statistical Package for the Social Sciences (SPSS) software (Version 25). Descriptive statistics, including frequencies and means, were employed to succinctly summarize the profiles and responses of the participating pharmacists. Applied inferential statistical techniques, such as chi-square tests and regression analysis, were used to examine the relationships between different variables. This approach yielded profound insights, significantly enhancing our comprehension of the attitudes and perceptions of pharmacists toward professional certification programs.

## 3. Results

**Figure 1:**
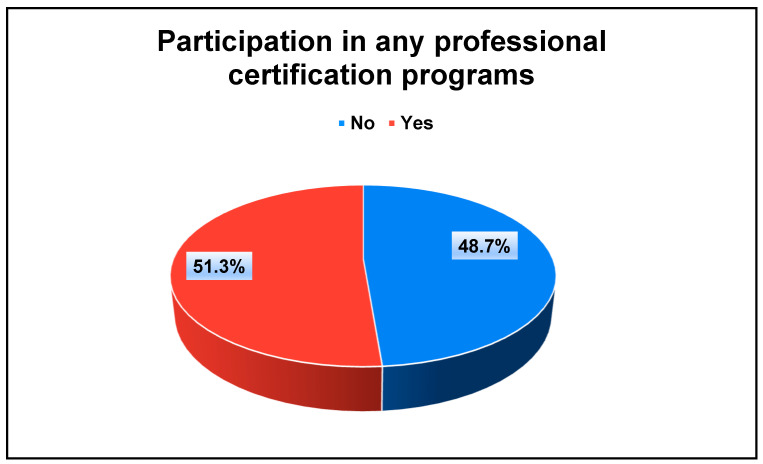
The distribution of the study participants regarding participation in any professional certification programs.

The cross-sectional survey was conducted among pharmacists in Saudi Arabia, with 394 participants meeting the inclusion criteria and completing the questionnaire. Table 1 provides a summary of the demographic characteristics of the respondents. The participants had a mean age of 27.97 years, with ages ranging from 22 to 69 years. The majority (74.1%) were between 18 and 30 years old. The gender distribution was balanced, with 51.3% male and 48.7% female. The average practicing period among the pharmacists was 4.2 years. Specifically, 69.5% had less than 5 years of experience, 20.8% had between 5 and 10 years, 6.1% had between 11 and 15 years, and 3.6% had more than 15 years of experience. Of the 394 participants, 48.7% were working in the Western Province, and 46.4% were employed in hospital settings. In terms of education, the highest pharmacy degree attained by the majority was a Doctor of Pharmacy (Pharm.D) (67.8%, 267), followed by a Bachelor of Pharmacy (B.Pharm) (18.3%, 72). Additionally, about half of the participants (51.3%) had previously engaged in professional certification programs, as shown in Figure 1.

### 3.1. Awareness among the Studied Participants

Table 2 provides a summary of the participants’ awareness regarding professional certification programs. A significant majority (73.1%) of respondents were aware of the availability of such programs for pharmacists in Saudi Arabia. The most common source of information was colleagues or peers (51.8%), followed closely by online searches (51.5%) and professional associations (35.8%). Regarding barriers to pursuing professional certification, participants rated pre-identified barriers on a scale from 1 to 5. The most significant challenges identified were a lack of awareness about available certifications (33% rated it as a significant barrier), financial constraints (32%), and time constraints (23.9%).

### 3.2. Participation in Certification Programs and Future Considerations:

Among those who had heard about professional certification programs, 55.3% were interested in joining programs in Clinical Pharmacy Specialties, 54.8% in Pharmacy Management and Leadership, 43.1% in Medication Safety and Quality Improvement, and 32.2% in Research and Academic Development in Pharmacy. The percentages of those interested in joining other programs are illustrated in Table 3. The impact of those programs on their careers is mentioned in Table 3. Among those who had heard about professional certification programs, 67.5% of participants reported having participated in at least one certification program. Of these, 21.8% were satisfied with their experience, while 15.2% reported being very satisfied. Additionally, 83% of participants expressed an interest in pursuing professional certification in the future, and 51.7% stated they would recommend pursuing professional certification to other pharmacists.

### 3.3. Certification Program Preferences and Access to Certification Programs

Table 4 outlines the participants’ preferences for assessment methods and the accessibility of professional certification programs. Half of the respondents (50%) favored short-term certification programs, while 42.1% preferred medium-term programs, and a smaller portion (7.9%) opted for long-term programs. Continuous evaluation throughout the program was preferred by 56.6% of the participants. Regarding accessibility, 43.7% felt that certification programs are geographically accessible for pharmacists in Saudi Arabia, and 33% considered the cost of these programs to be reasonable and affordable. 

### 3.4. Relations

There was no statistically significant relationship found between awareness among pharmacists and age and gender (*p* > 0.05). There was a statistically significant relationship between awareness among pharmacists and region of practice (*p* = 0.002) as awareness was significantly higher in the Western and Central Provinces. However, there was no statistically significant relationship between awareness among pharmacists and years of experience, primary practice setting, and highest pharmacy degree attained (*p* > 0.05). Also, there was no statistically significant relationship between gender and satisfaction with the certification process, pursuing professional certification in the future, recommending pursuing professional certification to other pharmacists in the future, and preference for professional certification programs (*p* > 0.05). 

## 4. Discussion

Participating in professional certification programs is a crucial way for professionals to stay up-to-date and advance their skills in today’s evolving field. These programs typically require passing exams set by organizations that uphold industry standards. Upon successful completion, professionals receive certificates that validate their expertise, experience, and competencies [10,11]. Our findings demonstrate that there is a notable level of interest among pharmacists in these programs, highlighting a general awareness of their importance for professional development. However, the actual participation rate is varied, suggesting that further efforts may be needed to increase engagement and involvement in these initiatives. In today’s job market, these credentials have become more important than ever, as many organizations prefer certified candidates for their credibility and the skills that they offer. This emphasis on hiring certified professionals can result in significant savings in costs, time, and effort for employers [12,13]. Professional certification offers a wide range of benefits that extend far beyond job opportunities. It plays a crucial role in career advancement, opening doors for promotions and providing financial incentives. These advantages are key motivators for individuals to pursue certification programs. In the healthcare sector, specialty certifications not only enhance the quality of patient care, but also strengthen professional credibility and reflect a deep commitment to the field. Certified professionals often feel more empowered, contribute more effectively to team efforts, and believe that their improved skills make a meaningful difference to patient outcomes. As such, it is essential for leaders to encourage their teams to pursue certifications, thereby elevating the overall expertise within the group [14,15]. Our findings provide valuable insights into pharmacists’ awareness, attitudes, and participation in professional certification programs, highlighting trends in pharmacy practice. We found that pharmacists in Saudi Arabia generally know about these programs, with higher awareness in the Central and Western Provinces compared to other regions. However, there is a need for better outreach to boost nationwide awareness. Interest in certifications, particularly in Clinical Pharmacy, Pharmacy Management and Leadership, and Medication Safety, is growing, and pharmacists recognize their value in enhancing their professional skills. Despite this, the level of actual participation remains lower than anticipated. Contributing factors may include insufficient institutional support, time limitations, and financial barriers. These challenges are not unique to pharmacists, since similar studies have noted that physicians and nurses face comparable issues that impact their professional education and certification [16,17].

Addressing these issues is crucial in fostering a culture of ongoing professional development in pharmacy. For instance, a study among nurses demonstrated that increased certification rates in perioperative units were significantly linked to reduced incidences of central line-associated bloodstream infections in surgical intensive care units [18]. Results from another study indicated that hospitals with a higher proportion of nurses holding baccalaureate degrees and certifications experienced lower 30-day mortality and failure-to-rescue rates among surgical patients [19]. There are a variety of reasons why people pursue professional certifications. For many, major motivating factors include a desire to improve patient care and career advancement. It was acknowledged that obtaining certification could result in increased pay and greater work possibilities [13]. These results are consistent with worldwide patterns, where certification is becoming more and more viewed as a means of advancing one’s career and gaining credibility in the healthcare industry [15]. Policy changes and educational reforms are needed to keep up with evolving pharmacy practices. Institutions should integrate professional certification into their training and support pharmacists in pursuing these opportunities. Awareness campaigns about the benefits of certification are also crucial. Increasing awareness, fostering positive attitudes, and promoting ongoing engagement in certification programs are crucial for pharmacists’ professional growth. To address challenges in pursuing certification, a multifaceted approach is needed. Financial aid like scholarships can make certification more accessible. Educational initiatives by pharmacy associations and schools can highlight the benefits of certification. Flexible learning options can help pharmacists manage their time better, and employers can motivate development by rewarding certification achievements. However, our study has some limitations. The reliance on self-reported data can introduce biases, and more research is needed to understand how certification affects pharmacists’ skills, practice outcomes, and patient care. A limitation of this study is the reliance on an online survey, which may have inadvertently excluded pharmacists with limited access to digital platforms, introducing potential sampling bias. We did not collect data on participants’ nationalities, which may affect interest in certification. Future research should examine how nationality influences engagement with certification programs, while also exploring the impact of different certification types on professional growth and addressing participation barriers. Additionally, expanding the range of available certification programs could address existing gaps and better support pharmacists’ professional development. Health authorities and academic institutions should work together to make certification programs more accessible, offer financial support, and incorporate these programs into ongoing education requirements. This would help encourage more pharmacists to participate and further their professional development.

## 5. Conclusions

Our study provides valuable insights into pharmacists’ awareness and engagement with professional certification programs. Although there is a general awareness and positive outlook on these programs, actual participation is limited by several barriers.

## Figures and Tables

**Table 1:** Demographic characteristics of studied participants.

Items	Study Participants (N = 394)	
	Number	Percentage (%)
**Age (Years)**		
** Mean (SD)**	27.97 (6.09)	
** Median (IQR)**	26.0 (24.0–31.0)	
**Range**	22–69	
**18–30 years**	292	74.1%
**31–60 years**	101	25.6%
**>60 years**	1	0.3%
**Gender**		
**Male**	202	51.3%
**Female**	192	48.7%
**Years of experience practicing as a pharmacist**		
Mean (SD)	4.2 (4.99)	
Range (Years)	0.0–33	
<5 years	274	69.5%
5–10 years	82	20.8%
11–15 years	24	6.1%
>15 years	14	3.6%
**Region of practice as a pharmacist**		
Western Province	192	48.7%
Central Province	110	27.9%
Eastern Province	50	12.7%
Southern Province	22	5.6%
Northern Province	20	5.1%
**Primary practice setting as a pharmacist**		
Hospital	183	46.4%
Community Pharmacy	102	25.9%
Pharmaceutical Companies	57	14.5%
Academia	30	7.6%
Pharmaceutical Manufacturing	14	3.6%
Others	8	2.2%
**Highest pharmacy degree attained**		
Doctor of Pharmacy (Pharm.D)	267	67.8%
Bachelor of Pharmacy (B.Pharm)	72	18.3%
Ph.D. in Pharmacy	26	6.6%
Masters in Pharmacy	19	4.8%
Residency	6	1.5%
Fellowship	2	0.5%
Residency and Fellowship	2	0.5%

**Table 2:** Awareness of professional certification programs among study participants.

Items	Study Participants (N = 394)	
	Number	Percentage (%)
**-Are you aware of the availability of professional certification programs for pharmacists in Saudi Arabia?**		
No	106	26.9%
Yes	288	73.1%
**-How did you become aware of these certification programs?**		
Online search	203	51.5%
Colleagues or peers	204	51.8%
Professional associations	141	35.8%
Employer or workplace	136	34.5%
Social media	5	1.3%
**-Please rate your agreement with the following statements** [Professional certification enhances career opportunities.]		
Strongly agree	191	48.5%
Agree	99	25.1%
Neutral	44	11.2%
Disagree	13	3.3%
Strongly disagree	47	11.9%
[Certified pharmacists provide better patient care.]		
Strongly agree	163	41.4%
Agree	108	27.4%
Neutral	58	14.7%
Disagree	21	5.3%
Strongly disagree	44	11.2%
[Certification leads to higher earning potential.]		
Strongly agree	148	37.6%
Agree	116	29.4%
Neutral	64	16.2%
Disagree	23	5.8%
Strongly disagree	43	10.9%
[Certification demonstrates commitment to the pharmacy profession.]		
Strongly agree	156	39.6%
Agree	100	25.4%
Neutral	68	17.3%
Disagree	18	4.6%
Strongly disagree	52	13.2%
[Professional certification improves skills and makes pharmacy practicing easier.]		
Strongly agree	174	44.2%
Agree	101	25.6%
Neutral	61	15.5%
Disagree	19	4.8%
Strongly disagree	39	9.9%
**-Please indicate the extent to which each of the following factors acts as a barrier to pursuing professional certification on a scale of 1 to 5, where 1 is not a barrier, and 5 is a significant barrier.** [Lack of awareness about available certifications]		
1	36	9.1%
2	42	10.7%
3	103	26.1%
4	83	21.1%
5	130	33.0%
[Time constraints]		
1	25	6.3%
2	49	12.4%
3	112	28.4%
4	114	28.9%
5	94	23.9%
[Financial constraints]		
1	20	5.1%
2	47	11.9%
3	110	27.9%
4	91	23.1%
5	126	32.0%
[Difficulty in finding relevant certification programs]		
1	40	10.2%
2	61	15.5%
3	102	25.9%
4	75	19.0%
5	116	29.4%

**Table 3:** Interest and perspectives on professional certification programs among study participants: preferences, future pursuits, and recommendations.

Items	Study Participants (N = 394)	
	Number	Percentage (%)
**Which of the following general categories of professional certification programs are you most interested in joining?**		
Clinical Pharmacy Specialties (e.g., pharmacotherapy, oncology)	218	55.3%
Pharmacy Management and Leadership	216	54.8%
Medication Safety and Quality Improvement	170	43.1%
Research and Academic Development in Pharmacy	127	32.2%
Health Technology and Informatics in Pharmacy	118	29.9%
Compounding and Dispensing Techniques	112	28.4%
Community and Ambulatory Care	96	24.4%
Pharmaceuticals Industries	2	0.5%
Project Management Professional (PMP)	1	0.3%
None	1	0.3%
**How likely are you to pursue professional certification in the future?**		
Very Likely	190	48.2%
Likely	137	34.8%
Neutral	61	15.5%
Unlikely	5	1.3%
Very Unlikely	1	0.3%
**How likely are you to recommend pursuing professional certification to other pharmacists in the future?**		
Likely	142	36.0%
Neutral	62	15.7%
Unlikely	4	1.0%
Very Likely	186	47.2%
**What format do you prefer for professional certification programs?**		
Blended (combination of in-person and online)	181	45.9%
In-person	105	26.6%
Online	108	27.4%

**Table 4:** Preferred assessment and accessibility of professional certification among study participants.

Items	Study Participants (N = 394)	
	Number	Percentage (%)
**What is your preferred duration for a professional certification program?**		
Short-term (e.g., a few weeks)	197	50.0%
Medium-term (e.g., a few months)	166	42.1%
Long-term (e.g., a year or more)	31	7.9%
**How would you prefer the certification program to be assessed?**		
Written exams	138	35.0%
Practical assessments	220	55.8%
Case studies or projects	143	36.3%
Continuous evaluation throughout the program	223	56.6%
**Do you believe that certification programs are geographically accessible to pharmacists in Saudi Arabia?**		
No	74	18.8%
Not sure	148	37.6%
Yes	172	43.7%
**Do you believe that the cost of certification programs is reasonable and affordable?**		
No	114	28.9%
Not sure	150	38.1%
Yes	130	33.0%
**Would you prefer it if professional certification programs were integrated with the continuous professional development (CPD) requirements for pharmacists?**		
No	57	14.5%
Not sure	97	24.6%
Yes	240	60.9%

## Data Availability

The corresponding author is willing to provide the data that supports this study’s findings upon receiving a reasonable request.

## References

[1] Professional Certifications and Occupational Licenses on JSTOR. https://www.jstor.org/stable/26743923.

[2] Rooney A.L., Paul M.P.H., Van Ostenberg R. Quality Assurance Methodology Refinement Series x x x Licensure, Accreditation, and Certification: Approaches to Health Services Quality. www.urc-chs.com.

[3] Burns A.L. (2014). Credentialing and privileging of pharmacists: A resource paper from the Council on Credentialing in Pharmacy. Consult. Pharm..

[4] 1SSCP—Learning. https://sscplearning.com/lms/.

[5] Al-Jedai A., Qaisi S., Al-Meman A. (2016). Pharmacy Practice and the Health Care System in Saudi Arabia. Can. J. Hosp. Pharm..

[6] Micallef R., Kayyali R. (2019). A Systematic Review of Models Used and Preferences for Continuing Education and Continuing Professional Development of Pharmacists. Pharmacy.

[7] Chapter 03 | Health | General Authority for Statistics. https://www.stats.gov.sa/en/1009.

[8] Al-Kubaisi K.A., Elnour A.A., Sadeq A. (2023). Factors influencing pharmacists’ participation in continuing education activities in the United Arab Emirates: Insights and implications from a cross-sectional study. J. Pharm. Policy Pract..

[9] Lenora G., Knapp P.D., James Kendzel M. (2009). ICE 1100 2010 (E)—Standard for Assessment-Based Certificate Programs.

[10] Saseen J.J., Grady S.E., Hansen L.B., Hodges B.M., Kovacs S.J., Martinez L.D., Murphy J.E., Page R.L., Reichert M.G., Stringer K.A. (2006). Future Clinical Pharmacy Practitioners Should Be Board-Certified Specialists. Pharmacother. J. Hum. Pharmacol. Drug Ther..

[11] Kandasamy G., Almaghaslah D., Almanasef M. (2023). An Evaluation of Continuing Medical Education among Pharmacists in Various Pharmacy Sectors in the Asir Region of Saudi Arabia. Healthcare.

[12] Adams P.S., Brauer R.L., Karas B., Bresnahan T.F., Murphy H. (2014). Professional Development Professional Development Professional Certification Its value to SH&E practitioners and the profession. International Handbook of Research in Professional 915 and Practice-Based Learning.

[13] Adeosun O.T., Adegbite W.M. (2023). Professional Certification and Career Development: A Comparative Analysis between Local and Foreign Certifications. Manag. Econ. Res. J..

[14] Gaberson K.B., Schroeter K., Killen A.R., Valentine W.A. (2003). The perceived value of certification by certified perioperative nurses. Nurs. Outlook.

[15] Schroeter K. (2015). The value of certification. J. Trauma Nurs..

[16] Frenk J., Chen L.C., Chandran L., Groff E.O.H., King R., Meleis A., Fineberg H.V. (2022). Challenges and opportunities for educating health professionals after the COVID-19 pandemic. Lancet.

[17] Shahhosseini Z., Hamzehgardeshi Z. (2015). The Facilitators and Barriers to Nurses’ Participation in Continuing Education Programs: A Mixed Method Explanatory Sequential Study. Glob. J. Health Sci..

[18] Boyle D.K., Cramer E., Potter C., Gatua M.W., Stobinski J.X. (2014). The relationship between direct-care RN specialty certification and surgical patient outcomes. AORN J..

[19] Blegen M.A. (2012). Does certification of staff nurses improve patient outcomes?. Evid. Based Nurs..

